# Techniques and Considerations in the Microfabrication of Parylene C Microelectromechanical Systems

**DOI:** 10.3390/mi9090422

**Published:** 2018-08-22

**Authors:** Jessica Ortigoza-Diaz, Kee Scholten, Christopher Larson, Angelica Cobo, Trevor Hudson, James Yoo, Alex Baldwin, Ahuva Weltman Hirschberg, Ellis Meng

**Affiliations:** 1 Department of Biomedical Engineering, University of Southern California, Los Angeles, CA 90089, USA; jortigoz@usc.edu (J.O.-D.); kscholte@usc.edu (K.S.); larsonce@usc.edu (C.L.); acobo001@gmail.com (A.C.); tqhudson@usc.edu (T.H.); james.yoo@usc.edu (J.Y.); abbaldwi@usc.edu (A.B.); aweltman1@gmail.com (A.W.H.); 2 Ming Hsieh Department of Electrical Engineering, University of Southern California, Los Angeles, CA 90089, USA

**Keywords:** Parylene, microfabrication, MEMS

## Abstract

Parylene C is a promising material for constructing flexible, biocompatible and corrosion-resistant microelectromechanical systems (MEMS) devices. Historically, Parylene C has been employed as an encapsulation material for medical implants, such as stents and pacemakers, due to its strong barrier properties and biocompatibility. In the past few decades, the adaptation of planar microfabrication processes to thin film Parylene C has encouraged its use as an insulator, structural and substrate material for MEMS and other microelectronic devices. However, Parylene C presents unique challenges during microfabrication and during use with liquids, especially for flexible, thin film electronic devices. In particular, the flexibility and low thermal budget of Parylene C require modification of the fabrication techniques inherited from silicon MEMS, and poor adhesion at Parylene-Parylene and Parylene-metal interfaces causes device failure under prolonged use in wet environments. Here, we discuss in detail the promises and challenges inherent to Parylene C and present our experience in developing thin-film Parylene MEMS devices.

## 1. Introduction

### 1.1. History and Types of Parylene

**Figure 1:**
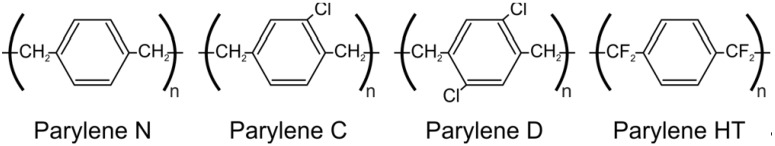
Chemical structure of Parylene types.

Parylene is the trade name for poly-(para-xylylene), a class of semicrystalline, hydrophobic polymers which can be deposited as thin, conformal, pinhole-free films using chemical vapor deposition (CVD). Parylene was discovered by Michael Mojzesz Szwarc in the late 1940s but was not commercialized until 1965 following the development of the Gorham deposition process at Union Carbide [1,2]. Types of Parylene that are commonly available include Parylene N, Parylene C, Parylene D and Parylene HT (Figure 1). A comparison of the properties of these films can be found in [3]. Parylene N is composed of an aromatic ring with attached methylene groups [4]; it has been used as a dielectric and has the slowest deposition rate of the Parylenes [5]. Parylene C features a single chlorine atom on its benzene ring and is recognized for its chemical inertness, electrical resistivity, low moisture permeability and proven biocompatibility. Parylene D has two chlorine atoms, resulting in similar properties to Parylene C with slightly higher temperature resistance. Parylene HT is a fluorinated variant of the Parylene N polymer and is marked by high temperature stability [6]. The two most common Parylene types in commercial applications are Parylene N and C, whereas Parylene C is the most common material encountered in MEMS devices.

Parylene C, referred from here on as Parylene, is a US Pharmacopeia class VI material [7], having the highest biocompatibility certification for a plastic material. For several decades, thin Parylene coatings were used to create waterproof insulation for electronics intended for use in harsh environments, a category that increasingly includes biomedical implants. Parylene exhibits low intrinsic stress in addition to optical transparency, mechanical flexibility and compatibility with several standard micromachining processes [8,9,10,11]. While Parylene can be used in combination with rigid substrates such as silicon and glass, it has been increasingly utilized as a flexible structural material in the growing field of polymer-based biomedical microelectromechanical systems (bioMEMS). As a dynamic polymer material, Parylene presents unique challenges during microfabrication and use which are further explored here.

### 1.2. Thin-Film Parylene Device Microfabrication

**Figure 2:**
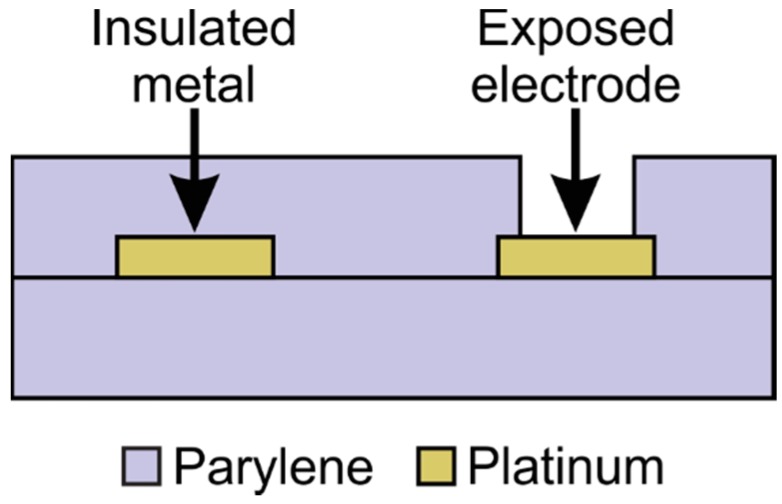
Cross section of a typical device showing insulated and exposed metal features, such as traces and electrodes, respectively.

Parylene-based bioMEMS are prepared through a combination of microfabrication processes and commonly consist of a simple sandwich design, with a base layer of Parylene, a thin layer of patterned metal defining traces and other components, and a top layer of Parylene. Figure 2 depicts a flexible device in which Parylene serves as both the structural material and insulator. However, devices may also be supported on standard rigid substrates such as glass and silicon when flexibility is not required. Regardless of the final format, microfabrication requires that Parylene be supported by rigid substrate during fabrication, with optional release of a free film device towards the end of the process.

Devices are realized using a combination of three process categories: additive processes (deposition), subtractive processes (etching) and patterning (e.g., photolithography). Parylene is deposited exclusively through a highly conformal CVD process at room temperature, with typical deposition rates on the order of ~2 μm/h. Other deposition methods are critical for polymer MEMS processing; spin coating is used to deposit photo-patternable polymer layers (photoresist) for use as an etch mask or shadow mask, or as a sacrificial layer, while physical vapor deposition (PVD) methods including evaporation and sputtering are used for metal deposition. Etching processes are limited to dry methods due to Parylene’s high chemical inertness. Reactive ion etching (RIE) and deep reactive ion etching (DRIE) are oxygen plasma-based techniques commonly used to etch Parylene. Parylene devices are predominantly patterned using standard UV lithography processes, and, more recently, nanoscale features can be patterned using electron beam lithography with appropriate protective layers [12] to prevent beam damage.

### 1.3. State-of-the-Art Parylene-Based Devices

**Figure 3:**
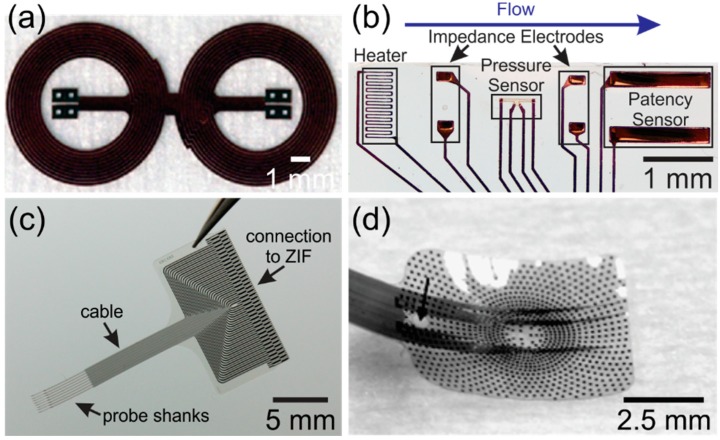
(**a**) Wireless coils (Reprinted from Reference [16] with permission from Elsevier); (**b**) flow, pressure and patency sensors to monitor hydrocephalus treatment (©2016 IEEE. Reprinted, with permission, from Reference [23]); (**c**) hippocampal neural probe array (©2017 IEEE. Reprinted, with permission, from Reference [37]); and (**d**) retinal prosthesis that matches the curvature of the eye (Reprinted from Reference [49] with permission from Elsevier). Parylene is transparent and the opaque features are metal.

Parylene is used extensively to coat printed circuit boards, wires, MEMS devices, and biomedical implants, and less commonly as a structural or substrate material for electronic and MEMS devices. Still, over the last several years, many devices for a large variety of applications have been described in the literature featuring Parylene as either the structural or substrate material (Figure 3). Parylene has been used as a flexible coiled cable to connect medical implants to external circuitry [13,14,15]. Radio frequency powered coils were fabricated with Parylene for wireless transmission (Figure 3a) [16,17] and more recently a new method to wirelessly transduce electrochemical impedance was developed using Parylene coils [18]. Parylene microsensors (Figure 3b) were developed to measure intraocular and intracranial pressures [19,20,21] and Parylene thermal sensors were created to detect small flows [22,23,24,25]. Other devices include neurocages for in vitro neural network study [26,27,28], bellows for drug delivery [29,30,31], an electrochemical patency sensor [32], microfluidic devices [7] and electrothermal valves [33]. In the field of neural prostheses Parylene was used to create both penetrating (Figure 3c) [34,35,36,37,38,39] and non-penetrating microelectrode arrays [40,41,42]. Microfluidic channels were integrated into Parylene neural probes to inject drugs [43,44,45]. Parylene-based cuff electrodes, which record neural activity within peripheral nerves, were also developed [46,47,48]. Parylene spinal cord stimulators have also been realized with high-density electrode sites [49,50].

Parylene is also a common material for sensory neural prosthetics, such as retinal implants. High-density electrode arrays were fabricated on Parylene that was subsequently thermoformed to match the curvature of the eye (Figure 3d) [8,49,51]. An origami-like device was fabricated to match the curvature of the eye without the use of heat [52]. Cochlear electrode arrays, which must conform to the complex anatomy of the inner ear, were developed by exploiting Parylene’s properties as a thin and flexible substrate [53].

Despite growing interest, many researchers report difficulties when processing Parylene MEMS, as well as various modes of material and device failure. These problems largely stem from a lack of well-defined protocols for the machining, use and handling of Parylene-based devices. Common processing techniques developed for semiconductor materials and glass are often incompatible with organic polymers, owing to Parylene’s limited thermal budget, gas permeability, low mechanical strength and unique chemical properties. While many of these obstacles are surmountable, solutions are rarely discussed in the literature. Here we present a compilation of common challenges encountered during the construction of Parylene MEMS and a description of current best practices to avoid these issues.

## 2. Challenges

### 2.1. Thermal Budget

**Figure 4:**
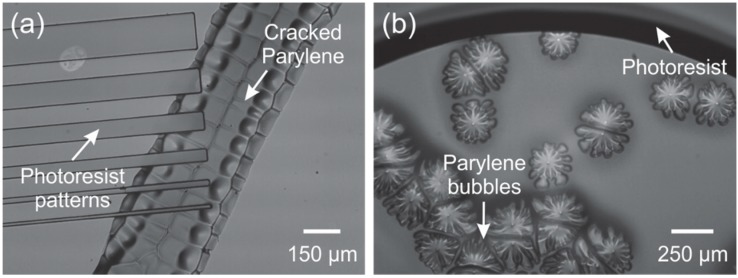
Images of Parylene damage due to high heat or radiation exposure. (**a**) Cracked Parylene after development and soft baking at 115 °C for 3 min; and (**b**) air bubbles appeared under the film, during hard bake at 90 °C for 15 min, in regions previously exposed to UV light during lithography.

One of the most persistent challenges of Parylene is its limited thermal budget, a consequence of its thermoplastic nature; in atmosphere Parylene is subject to oxidation at temperatures greater than 100 °C, glass transition temperature around 90 °C and a melting temperature at 290 °C [10]. These temperatures are commonly encountered and even surpassed during standard silicon micromachining and electrical packaging. For example, during photolithography, photoresist must be soft-baked at elevated temperatures (100–120 °C) to remove residual solvent and exothermic reactions during the UV activation of the resist can generate temperatures of up to 200 °C [54]. Soldering, plasma etching, PVD and other methods common to micromachining require temperatures which can cause Parylene to burn, bubble, or crack (Figure 4).

**Figure 5:**
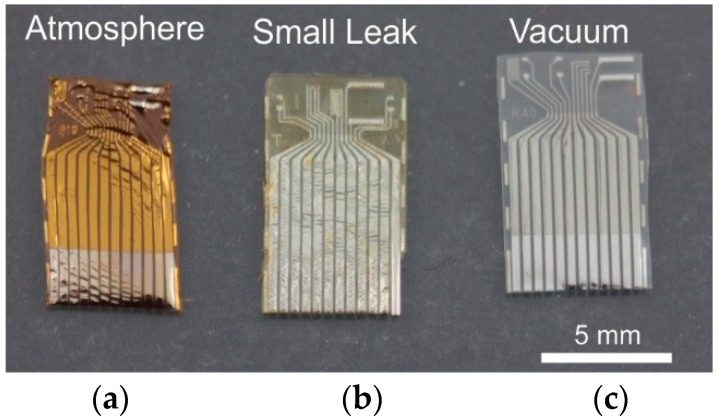
Comparison of thermal annealed devices in the presence of oxygen (**a**,**b**) and under intact vacuum (**c**). Devices were heated at 200 °C for 48 h.

Thermal annealing is a common treatment for improving adhesion between Parylene-Parylene interfaces [55,56]. This process requires high temperature (>200 °C) compared to Parylene’s glass transition temperature and must be performed under vacuum to avoid the effects of oxidation (browning, wrinkling and becoming brittle) as shown in Figure 5.

### 2.2. Water Diffusion through Parylene Films

Parylene is employed as encapsulation for medical implants, such as stents, pacemakers and neural probes [57,58] due to its strong water barrier properties. However, like all polymers, Parylene is in fact permeable to moisture and gases [59]. Both water and ions in solution (i.e., salt) can diffuse through thin layers of Parylene in less time than the intended use duration. For a Parylene-based bioMEMS or microdevice with insulation layers only a few microns thick, limiting permeation is critical to prevent electrical shorts, corrosion of encapsulated components and catastrophic failure. The most effective method at preventing water permeation is the use of thermal annealing to increase the crystallinity, though this only serves to slow permeation, not prevent it outright [56,60]. Unfortunately, there is scarce data quantifying the scale of the problem, particularly for the very thin (~µm) films used in Parylene-based microdevices. Below we describe some observations and measurements we have collected on the topic.

#### 2.2.1. Water Permeability

Published values for water vapor transmission rate (WVTR) in Parylene can vary quite substantially between sources. Specialty Coating Systems (SCS) [61] and Para Tech [62], two prominent vendors for Parylene coatings, publish WVTR values of 0.0830 and 0.0550 g·mm/m^2^·day, respectively, at 37 °C. Menon et al. [63] measured water vapor permeation in thin, small-area Parylene structures for both annealed and un-annealed films; WVTR values calculated from reported permeation measurements are listed in Table 1. WVTR values may differ based on deposition parameters and may be non-linear with film thickness for very thin films, as transmission through defects or large pores in thin-films may dominate over diffusion-driven transmission.

In Table 1, we provide our own WVTR measurements for annealed and unannealed films of varying thicknesses. Films were deposited on planar silicon wafers using a PDS Labcoter 2010 (SCS, Indianapolis, IN); annealed films were heated for 48 h at 200 °C under vacuum. The measurements were based on the beaker method for determination of water vapor permeability [64]. Glass beakers with ~40 mL of deionized water were sealed with Parylene (1.7 cm diameter circular aperture) using marine epoxy. The beakers, kept at room temperature and approximately 33% relative humidity, were weighed weekly for 2 months (mean values recorded in Table 1).

Notably, our values differ from Menon et al. despite similar temperatures and humidity. In agreement with Menon et al. we see a significant decrease in WVTR values after annealing films; for 10 and 15 µm thick films we see a 25% decrease in WVTR, in general agreement with reports and anecdotes from researchers noting a decrease in water permeation and an increase in effective lifetime for annealed Parylene/Parylene-coated devices. There was no significant decrease in WVTR for the annealed 5 µm film, which indicate that films at this thickness have permeation driven by defects rather than diffusion through the bulk.

#### 2.2.2. Ion Permeability

Parylene is known for having excellent ionic barrier properties, particularly when compared to other polymers. Under salt-fog tests (ASTM B117-(03)), SCS reported no evidence of corrosion or salt deposits on Parylene-coated PCB boards after 144 h of exposure [61] and Mordelt et al. reported 25 µm thick layers of Parylene withstood 0.9% saline solution for up to 30 days before breakdown [65]. Very thin layers of Parylene (<10 µm), however, are still susceptible to ion intrusion over relatively short time periods. In many Parylene-based microdevices, ionic intrusion into a Parylene insulation layer can change its dielectric properties, thereby increasing parasitic coupling between lines or decreasing shunt impedance [66,67]. In this manner ion permeability may affect device performance even if ions never breach the Parylene barrier. We observed that the use of thermal annealing can lower the rate of ion diffusion across a thin Parylene member but have not yet quantified the effect.

### 2.3. Delamination/Adhesion

**Figure 6:**
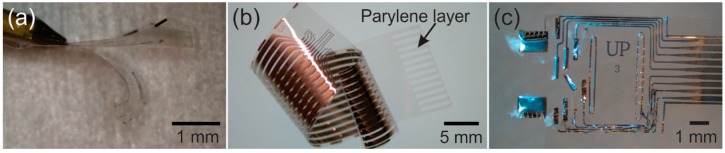
Parylene devices where delamination was noticeable on the macro scale. (**a**) Parylene layers visibly split from each other; (**b**) detachment of the top Parylene layer from the metal and bottom Parylene layers; and (**c**) electrodes and metal traces began to move or slide around between the Parylene layers.

Parylene suffers from poor adhesion to itself and noble metals, such as gold and platinum, a considerable drawback in the implementation of Parylene for bioMEMS. The adhesion of Parylene devices, which consist of thin films of Parylene-metal-Parylene sandwiches, is compromised when devices are soaked in wet environments [58,60,66,68,69]. Weak adhesion can accelerate catastrophic failure of Parylene devices; as Parylene films lift off a substrate, voids form in which water vapor can condense, creating continuous paths of solution that create electrical shorts and drive further delamination (Figure 6). Several strategies were investigated to improve adhesion of Parylene to substrates such as silicon and glass, including melting, anchoring, surface roughening, thermal annealing, surface plasma treatment and the inclusion of chemical layers, such as silane A-174 and plasma polymerized adhesion layers [70,71,72] but many of these techniques are not applicable or calibrated for adhesion of Parylene to Parylene, or thin-film metals used in polymer MEMS and flexible electronics.

Adhesion between two surfaces is achieved through chemical and physical interventions. In the case of Parylene-Parylene interfaces, adhesion is dominated by physical adsorption, whereas for Parylene-metal interfaces, adhesion is typically a combination of hydrogen bonding and Van der Waals forces [56]. Poor adhesion between Parylene and other materials may result from differences in surface energy at the interface [73] between hydrophobic Parylene and hydrophilic metals, a phenomenon exacerbated by surface contamination. The presence of internal stress between layers [60] appears to further aggravate adhesion failure and hasten delamination. Delamination under wet conditions may be induced by water vapor condensing within voids created by surface particulates present during CVD [74], suggesting that surface cleanliness is critical in maximizing adhesion. Annealing of Parylene (48 h at 200 °C under vacuum) improves adhesion. Reports suggest that annealing increases the entanglement of the polymer chains while reducing stress by recrystallization of Parylene-Parylene interfaces [57,75]. Interposer layers have also been investigated to either modify the surface energy of a coated material, thereby improving chemical adhesion, or to create a barrier against water vapor, minimizing water intrusion and subsequent failure [76,77,78]. Plasma-enhanced Parylene is a method to improve adhesion of Parylene to other surfaces by cleaning and modifying the film within the process chamber [79]. For a detailed study of methods to improve adhesion of Parylene layers and to platinum, the reader is referred to [56].

**Figure 7:**
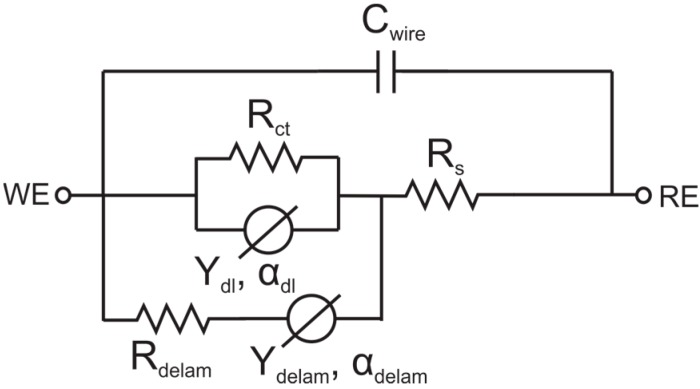
Circuit model of a Parylene-metal-Parylene device with an exposed electrode under chronic soaking conditions. *R*_ct_ represents the charge transfer resistance at the exposed electrode surface, while *Y*_dl_ models the double-layer capacitance at the electrode-electrolyte interface as a constant phase element. *R*_delam_ and *Y*_delam_ represent the resistive and capacitive charge transfer through the Parylene insulation; the magnitude of *R*_delam_ decreases as Parylene-Parylene delamination progresses. *R*_s_ represents solution resistance; *C*_wire_ represents parasitic capacitance; WE represents working electrode/exposed electrode; RE represents reference electrode.

Delamination at either the Parylene-Parylene or Parylene-metal interface can affect device performance well before catastrophic failure occurs. Many Parylene devices rely on insulated traces which terminate in exposed electrodes; delamination can alter this conductive pathway and lead to signal drift. These changes can be observed by monitoring the electrical/electrochemical impedance of an electrode and trace insulated in Parylene and immersed in water and understood using a modified Randles circuit model [80] (Figure 7). For a perfectly insulated electrode, the circuit consists of a solution resistance *R*_S_, charge transfer resistance *R*_ct_ and a constant phase element *Y*_dl_ to represent the double layer capacitance. Delamination presents an alternate conduction path from the electrode to the solution, with *R*_delam_ representing resistance through the insulation and constant phase element *Y*_delam_ representing the capacitance through the insulation between electrode trace and electrolyte. A capacitance *C*_wire_ is also included to model parasitic capacitances within connecting cables, which causes a phase roll-off at high frequencies.

**Figure 8:**
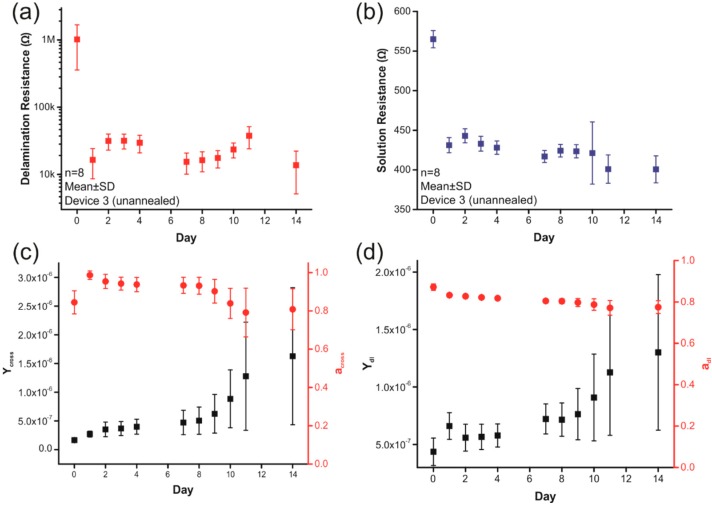
The results of modeling electrochemical impedance spectroscopy (EIS) spectra between a thin-film platinum electrode insulated between 10 μm Parylene layers and a large platinum counter electrode during a 14-day soak in 1× phosphate buffered saline (PBS) at 37 °C. Both (**a**) *R*_S_ and (**b**) *R*_delam_ drop after the first day, while the magnitudes of both (**c**) the cross-insulation capacitance (*Y*_cross_) and (**d**) the double layer capacitance (*Y*_dl_) steadily increase over the course of the test.

This model was confirmed using electrochemical impedance spectroscopy (EIS) data collected from platinum electrodes, coated and insulated with Parylene C films and soaked in 37 °C saline for 14 days (*n* = 8 electrodes) (Figure 8). All electrodes measured 300 × 1500 µm^2^ and were constructed from 2000 Å thick platinum insulated between 10 µm thick Parylene layers on the same die. EIS measurements were recorded between each electrode and a large platinum counter electrode using a Gamry Reference 600 potentiostat (Gamry Instruments, Warminster, PA, USA), with an Ag/AgCl electrode used as a reference. Modeling results show that both solution resistance and delamination resistance drop dramatically after only one day of soaking, while the interface capacitances gradually increase in magnitude over the testing period. The dramatic, two order-of-magnitude drop in delamination resistance may be due to either water penetration through the Parylene or the early stages of delamination and the small drop in solution resistance may be due to delamination around the exposed electrode, which would increase the electrode’s effective surface area.

### 2.4. Packaging and Electrical Connections

**Figure 9:**
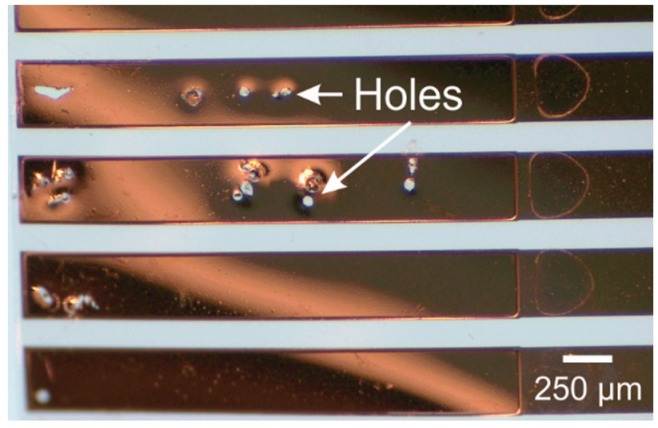
Holes left by gold removal during ultrasonic wire (ball) bonding on a gold thin film on Parylene substrate.

Electrical packaging of thin film electrodes (typically 100–200 nm thick) on free film Parylene-based devices is exceptionally challenging. Numerous problems arise from the thin profile, flexibility and limited thermal budget of Parylene films. Notably, Parylene is fundamentally incompatible with soldering, due to the high temperatures required (typically 250 °C) and the low glass transition temperature of Parylene (~90 °C) [81]. Similarly, Parylene devices are poorly compatible with conventional wirebonding, which makes it difficult to package Parylene devices with silicon integrated circuits. Ball bonding typically requires temperatures above 300 °C to achieve thermocompression [82], risking thermal damage to Parylene, and even when attempted the resulting bond between the wire and the thin film on the Parylene detaches when the tool retracts, since the metal-metal bond is stronger than the metal-Parylene bond (Figure 9).

Batch and reversible electrical connections to Parylene devices can be achieved using zero-insertion force (ZIF) connectors (Hirose Electric Co., Ltd., Tokyo, Japan), which are hinged friction-force connectors with spring-like copper pins that contact targeted pads when closed. Designed originally for connecting flat flexible cables (FFC) to PCB, ZIF connectors can be made compatible with Parylene electronics by mounting Parylene devices on thicker sections of more rigid polymers such as polyetheretherketone (PEEK). While simple, reversible electrical connections are possible, the contact pads have large, fixed footprints compared to flip-chip or wire-bonding. Though ZIFs are designed for repeated connections, it should be noted that after approximately 15 cycles, connections can fail due to wear and tear through the Parylene [83] substrate by the mechanical pins.

**Figure 10:**
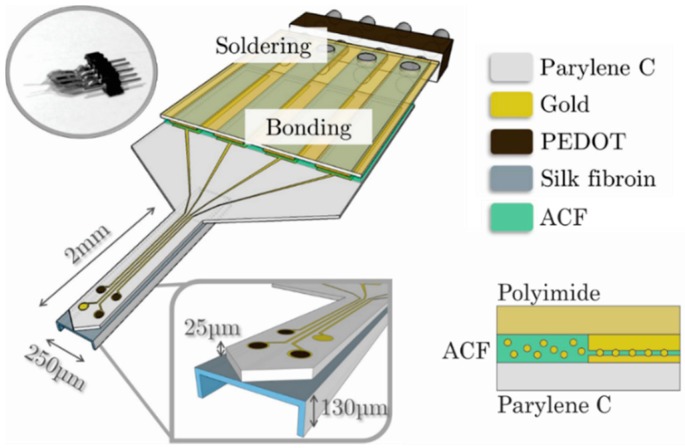
Schematic representation of a Parylene-based neural probe consisting of three PEDOT (poly(3,4-ethylenedioxythiophene))-nanostructured electrodes and one gold electrode as control. The device is anisotropic conductive film (ACF) bonded onto a flexible polyimide cable, which is then soldered onto a pin connector adapted to the wireless acquisition system. The cross-section shows bond pads from the device bonded via ACF to the bond pads of the polyimide cable. Reprinted from [88] with permission from Elsevier.

Other methods include the use of conductive epoxies [84] or adhesive films. Anisotropic conductive film (ACF), a common material for semiconductor packaging, comprises a thin resin of suspended polymer spheres which form a unidirectional conductive path under mild heat and pressure. ACF curing temperatures are relatively high (150–200 °C) but oxidation of Parylene can be avoided by applying the film under an inert atmosphere, or lowering the curing temperature and adjusting the time required to cure. For high bond yield, alignment between the substrates is important and in the case of building a custom jig or adapting an existing flip chip bonder, the levelness and thermo-mechanical properties of the bond head are critical. In this way, ACF can be successfully used for electrical packaging to Parylene devices including ASIC integration (Figure 10) [85,86,87,88]. In unpublished results, we achieved large-area packaging of Parylene electronics with pitch and width of contacts down to (100 µm pitch) using ACF (Dexerials CP13341-18AA, Dexerials, Tokyo, Japan).

Alternatively, another strategy is to directly incorporate integrated circuits with Parylene devices during the fabrication process to achieve the densest connections. Rodger et al. developed a method to form interconnects between Parylene devices and bare silicon integrated circuit dies during wafer-level processing, by etching through-holes in a carrier wafer, affixing the dies with temporary adhesives, and then evaporating metal. But because thin-film metal is deposited directly on top of the inserted chip, this method is extremely sensitive to the planarity of the dies [89]. Chang et al. developed a method to spread conductive epoxy over a Parylene layer to create interconnects to a silicon die, which was held in place by a PDMS mold, however, this method was prone to shorts between bond pads resulting from bridging by the low viscosity epoxy [90]; this required selective repair using laser ablation.

### 2.5. Sterilization

Parylene coatings for biomedical devices and Parylene-based bioMEMS require sterilization before use in vivo. However, many standard sterilization protocols require application of high heat or oxidative stress, which can damage or otherwise change Parylene bulk properties. For example, an autoclave creates a high temperature and high humidity environment for sterilization, which can cause Parylene bilayers to delaminate and Parylene coatings to lose adhesion [57,91]. Alternative methods, such as the use of ethylene oxide, have been used successfully without damage to thin-film Parylene devices [36]. Several methods for sterilizing Parylene devices are described in literature and a brief summary of various methods and the major conclusions drawn regarding the effect on Parylene, is compiled in Table 2. Notably, steam autoclaving, one of the most common methods, is also one of the most destructive. Other potentially damaging methods include electron beam sterilization, which can cause ionization and a decrease in crystallinity of bulk Parylene and gamma sterilization, which appears to decrease the adhesion between Parylene and metal.

We previously examined the use of hydrogen peroxide plasma as a means to sterilize Parylene-based electrochemical sensors. Devices were visibly unchanged following treatment and electrode characterization, using EIS and cyclic voltammetry, indicated no changes in electrode properties following treatment [32].

### 2.6. Handling

Finally, we note that owing to the thin and flexible nature of Parylene devices, they can be potentially damaged by rough handling. Electrical traces incorporated into Parylene devices or ribbon cables are actually surprisingly robust, able to survive down to a bend radius of 100 µm and under fatigue testing of up to 100,000 bends [76]. However, we observed that wrinkling or crumpling of Parylene devices, which can happen inadvertently, can cause destructive creases in thin metal connections. Parylene devices are also very light and can easily be blown away by the nitrogen streams commonly used for cleaning microdevices, or by the venting of a vacuum chamber, or even by exhalation. They are also subject to strong static forces, owing to Parylene’s properties as an electrical insulator. We recommend handling Parylene devices gingerly, with tweezers, held by the edge of the device or even by a purpose built Parylene tab designed into the structure. Weakly adhesive double-sided tape can be used to secure Parylene devices temporarily, during post-processing, packaging or imaging.

## 3. Micromachining of Parylene Films

Even though processes for silicon were successfully adapted to micromachine Parylene, there is a lack of well-defined protocols and standards when working with this polymer. Parylene-based MEMS and microdevices are typically constructed using a combination of bulk and surface micromachining and photolithography. In a typical process flow, a foundational Parylene layer is deposited on a support substrate, almost always a silicon wafer with its native oxide layer intact, using CVD. Subsequent layers of metal or polymer are then deposited and patterned using photolithography. Metal layers commonly serve as conductive traces or electrodes and are deposited using evaporation or sputtering and patterned by lift-off. Additional polymer layers include additional Parylene films, which may be patterned on-top of sacrificial photoresist patterns to create three-dimensional structures. Structures may be created using O_2_ plasma etching through a photoresist mask, to create MEMS components using bulk-micromachining, or to expose metal electrodes covered by polymer insulation. Finally, the complete device is removed from the support substrate and packaged. Below we describe challenges and solutions encountered during each step of fabrication for Parylene-based microdevices.

### 3.1. Deposition

**Figure 11:**
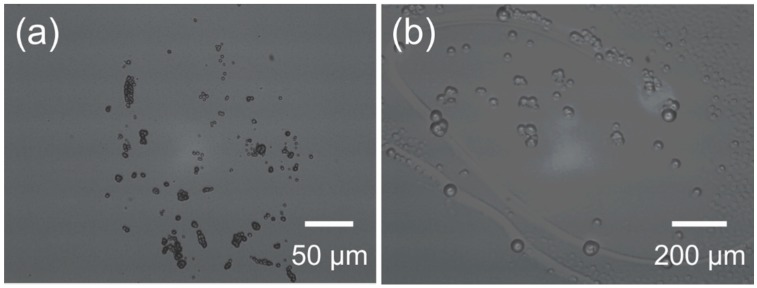
(**a**) Deposited Parylene layer incorporating spherules which results in a cloudy in appearance when observed by eye; and (**b**) magnified photograph revealing the presence of small clusters of spherules.

Parylene is deposited by CVD, producing a highly uniform and conformal coating [103]. Typically the film is transparent and homogenous; however, in some instances Parylene coatings may be marred by odd “spherule” inclusions. The macroscopic appearance is hazy and white, sometimes described as “cloudy” Parylene. The microscopic appearance is presented in Figure 11. The spherules may be unreacted Parylene monomers that bond to each other in the gas phase prior to deposition, a result of insufficient molecular collisions before deposition caused by a high volume-to-surface-area of the deposition chamber [104]. Increasing the surface area of the chamber, by including structures with large surface-area such as a mesh, may prevent the formation of these spherules. SCS, a manufacturer of Parylene coating tools, describes the cause as high deposition rates and chamber pressure.

This phenomenon was repeatedly observed when coating Parylene-based devices that were previously subjected to some form of mechanical action, such as ultrasonic treatment or scrubbing during a metal-liftoff process. These devices, when insulated with CVD Parylene, frequently present with this cloudy appearance, with the densest appearances of spherules along the edges of thin-film metal structures or at locations where the mechanical action was most severe. It is unclear whether these phenomena interfere with adhesion between the Parylene and other layers and whether they impact the integrity of the Parylene coating.

### 3.2. Lithographic Processes

Microfabrication commonly entails photolithographic patterning of photoresist to serve as an etch mask, lift-off mask, or patterned sacrificial layer. Photolithography involves a series of sub-processes, such as coating, pre/post baking, UV light exposure and development. These processes involve the use of heat and UV radiation and all can compromise the integrity of Parylene films.

**Figure 12:**
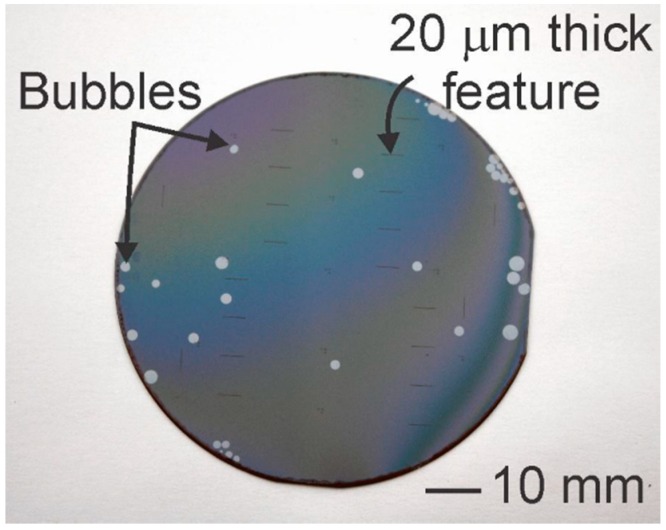
Observed gas bubbles in a Parylene coated wafer following UV exposure of a photoresist coating.

For example, the combination of Parylene gas permeability and the off-gassing of photoresist during UV exposure [54] can lead to the formation of bubbles in Parylene film. Figure 12 shows a representative image of this phenomenon, following UV exposure of a 20 μm thick positive resist layer on top of a 10 μm thick Parylene film. The phenomenon tends to be more pronounced with thicker resists and higher exposure dosage of UV radiation.

Photodegradation of Parylene has been reported in literature through a two-step process involving direct photolytic processes resulting in the formation of UV and IR absorbing structures, followed by photo-induced oxidation of the methylene groups and benzene ring [105]. It is also well known that UV radiation can deteriorate the thermal and electrical properties of Parylene films if the doses are large (>12 J/cm^2^) [106]. Although the small doses of UV exposure during lithography are unlikely to reach the threshold for full photodegradation, it is hypothesized that some combination of minor oxidation and mechanical/thermal stress may be responsible for the observed phenomenon when thick resist films are used. For example, bubbles between the Parylene and substrate may appear following the UV exposure step for photolithography (Figure 12). Piercing the Parylene bubbles in select non-critical areas and the use of a vacuum (after piercing) was found to aid in removal of the bubbles.

Photolithography has long been implemented and thoroughly characterized on silicon and glass wafer substrates. In adapting traditional microfabrication techniques to Parylene substrates, photoresist coating protocols for silicon have largely been ported over and calibrated on a case-by-case basis. In order to provide a practical guide for achieving desired photoresist thickness on Parylene and to better characterize the process, spin curves were created for two different photoresists on Parylene-coated silicon substrates and compared to those for silicon and glass substrates. Spin curves were produced for both AZ P4620 (Integrated Micro Materials, Argyle, TX, USA), a common etch mask and sacrificial layer resist and AZ 5214E-IR (Integrated Micro Materials, Argyle, TX, USA), a common lift-off resist. The spin-coating parameters used are listed in Table 3. Five 100 mm silicon wafers coated in 8 µm of Parylene were coated with photoresist at spin rates from 1000 rpm to 5000 rpm in 1000 rpm intervals. Five 100 mm prime silicon wafers and glass wafers were also coated at each spin rate for comparison. 5 mm-wide strips of photoresist near the wafer center and wafer edge were swabbed away from the surface using acetone and thickness was measured at these points using a surface profilometer.

**Figure 13:**
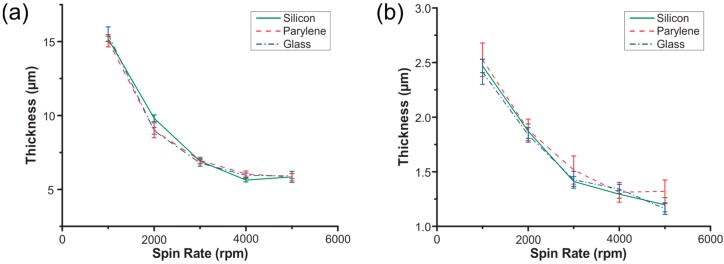
Spin curves for (**a**) AZ P4620 and (**b**) AZ 5214E-IR resists on silicon, glass and Parylene. Error bars represent standard deviation (*n* = 5).

To compare the spin curves of different surfaces, thicknesses were plotted against ω*_rpm_*^−½^ and least-squares linear regression was performed on each set, followed by testing for coincidence of the regression lines (Figure 13). No significant difference was found among the spin curves on Parylene, silicon and glass with AZ P4620, nor with AZ 5214E-IR (*p* < 0.05). These spin curves may serve as a starting point in the practical design of a Parylene microfabrication process.

### 3.3. Metal Deposition

Metal deposition is a key process in the fabrication of polymer MEMS devices to create conductive elements such as traces and electrodes. The deposition of metal on Parylene, however, is rife with complications. Both sputtering and evaporation can induce significant intrinsic and extrinsic stress which can induce curvature in the resulting free-film Parylene devices. In addition, the deposition of high melting point metals, such as platinum, through high temperature processes, such as evaporation, can lead to cracking of the metal film or the underlying Parylene due to thermal stress, a result of mismatch in the thermal coefficients of expansion and film stress.

**Figure 14:**
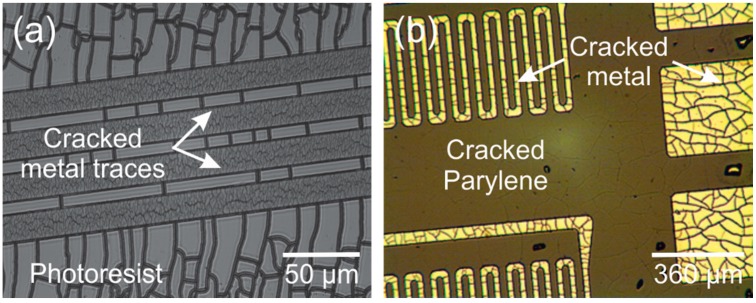
(**a**) Stress-induced cracking of deposited platinum likely due to excess heat generated during the process; and (**b**) cracked metal traces after lift-off.

Figure 14 shows an image of platinum (2000 Å) deposited on a Parylene coated silicon wafer (10 µm thick Parylene film), with stress induced cracks. The platinum was evaporated with a Temescal BJD-1800 e-beam evaporator using an uncooled stage; during evaporation wafer temperature surpassed 110 °C as measured with temperature monitor stickers (Omega Engineering, Norwalk, CT, USA) placed on the back of the wafer. Changes in the deposition rate and tool power were insufficient to prevent cracking. Improving the thermal contact between the wafer and the stage is difficult, in part because the Parylene which coats the back of the silicon wafer serves as an insulator. Breaking the deposition into a series of four steps with 15 min pauses between each deposition can prevent cracking with metals such as titanium, evaporated at a lower temperature but is insufficient for platinum. Ultimately, the most reliable method to avoid cracking was to use a tool (CHA Industries MARK 40, Fremont, CA, USA) with a larger throw distance between the metal target and the wafer stage (22″ compared with 8″ for the Temescal) and depositing the platinum in four 500 Å steps. Heat was measured with temperature monitor stickers as previously described and was maintained below 77 °C.

**Figure 15:**
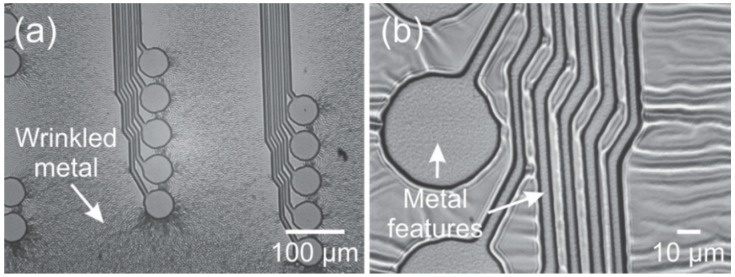
(**a**) Severe wrinkling/rippling seen in sputtered deposited platinum film results from compressive stresses of a higher magnitude; and (**b**) wrinkled areas around the device metal features.

Depositing metal through sputtering can avoid thermal stress and cracking but can impart severe film stress, warping the Parylene film and forcing released devices to curve. Figure 15a is an image of a Parylene substrate patterned with a negative profile photoresist for metal lift-off (AZ 5214E-IR) and sputter coated with 2000 Å of platinum. The rippled appearance is typical of Parylene films coated by sputtering and highlights the severity of film stress (Figure 15b).

**Figure 16:**
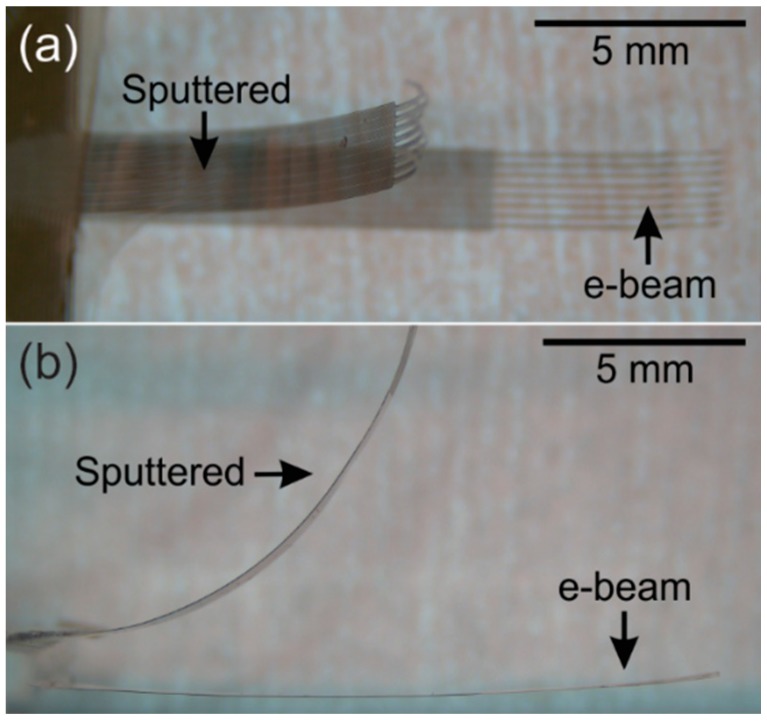
(**a**) Top view; and (**b**) side view of neural probe arrays after release from silicon carrier wafer. Arrays fabricated with sputtered platinum possessed more severe curvature than with platinum deposited by e-beam.

Figure 16 shows a micromachined Parylene-based neural probe array prepared with sputtered platinum. The high level of intrinsic stress (estimated as 510 MPa provided by LGA Thin Films, Santa Clara, CA, USA) forces the final device to curve severely, rendering it unusable. By comparison, the same devices are shown prepared with e-beam evaporated platinum, demonstrating reduced curvature.

### 3.4. Etching

Due to the inertness of Parylene it is not practical to etch it chemically. Although there are reports of wet etching Parylene using chloronapthelene or benzoyl benzonate at 150 °C [107], this is an extreme temperature for Parylene and can affect the bulk properties of unetched sections. Thus, mechanisms to selectively etch Parylene are limited to dry techniques. Parylene can be removed with oxygen plasma etching [108,109,110,111], reactive ion beam etching [112], oxygen reactive ion etching (RIE) and a deep reactive ion etching (DRIE) like method involving alternating cycles of oxygen plasma etching and fluorocarbon passivation layer deposition [108,112,113,114,115,116]. The latter method is referred to as O_2_ DRIE.

Photoresist etch masks are most commonly used to lithographically pattern Parylene, despite the low selectivity (approximately 1:1). Other materials including some metals can be used as masking materials if a high etch rate and good selectivity are required [115] but the coating and etching of metal masks may induce many of the thermal mismatch problems described above. Additionally, re-deposition of metal during the etch process is common and as such photoresists remain the most common choice [116,117]. Across a broad range of processes, AZ P4620, a positive-tone photoresist, was used as a Parylene etch mask for both O_2_ RIE and an O_2_ DRIE process (presented in [113]) to etch a variety of structures into Parylene film, ranging from 1 to 15 µm deep. Selectivity rates typically vary between 0.9 and 1, for etch rates varying between 0.55 and 0.80 µm/min.

**Figure 17:**
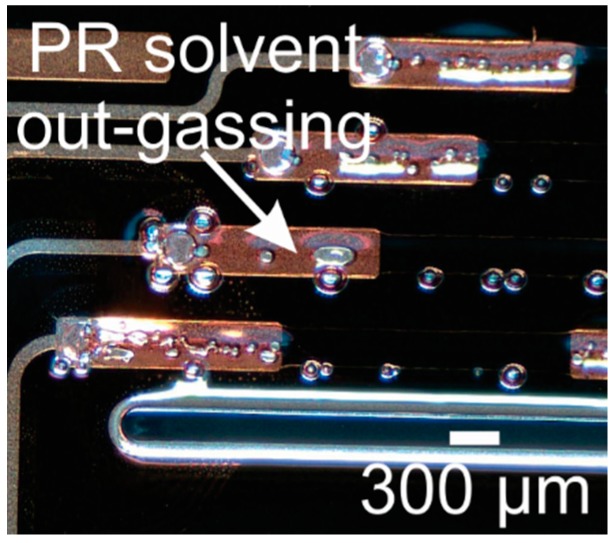
Out-gassing of photoresist solvent results in bubbling observed in sacrificial photoresist structures sandwiched in Parylene following O_2_ deep reactive ion etching (DRIE).

The most common problem we observe during plasma etching of Parylene is the formation of gas bubbles, either within photoresist layers or between the support substrate and Parylene coating. The apparent cause is the off-gassing of volatile components of photoresist or remnants of the solvent used to distribute the photoresist. Minor off-gassing from photoresists may be inconsequential, or even unnoticeable, when processing on rigid substrates, however Parylene is gas-permeable and even minor volatile products can produce bubbling when under vacuum. Bubbles may deform photoresist masks, creating holes which lead to etch damage, or deform the Parylene itself making future processing difficult. Figure 17 shows a representative example photoresist bubbling observed following a O_2_ DRIE using 80 W RF and 900 W ICP power. This phenomenon occurs more readily when using high power, high density, inductively coupled plasmas. Very thick photoresist layers (>10 µm) and, in particular, very thick edge beads are more susceptible, likely due to the residual solvent not driven off during soft-baking steps. We also observed the appearance of gas bubbles when processing multi-layer Parylene devices featuring three-dimensional Parylene structures defined using sacrificial layers. This is a relatively common technique where photoresist is patterned and then coated in Parylene to create Parylene-based microfluidic or mechanical structures. These sacrificial layers are particularly susceptible to forming bubbles during subsequent etch steps, as gas expands rapidly under the low pressure required for plasma etching, faster than the rate of diffusion through the Parylene film.

The formation of these gas bubbles appears particularly sensitive to the hard-baking step following photoresist development. In a simple experiment, Parylene coated 4″ silicon wafers were masked with 10 µm thick AZ P4620 and, following development, hard-baked on a hot plate at 90 °C for either 0, 2, or 12 h. Following a brief O_2_ DRIE process (approximately 2 µm etch), we observed catastrophic bubbling for the sample baked for 2 h, with minimal or no bubbling for unbaked samples and samples baked for 12 h. We hypothesize that after 2 h of hard baking, volatiles diffuse through the Parylene layer and become trapped between the Parylene and silicon wafer substrate once the wafer cools. We suspect these volatiles are responsible for later bubbling in the DRIE. By contrast, 12 h of hard baking may completely drive off photoresist volatiles from the wafer, while omitting the hard bake entirely prevents any diffusion of volatiles under the Parylene. We strongly recommend avoiding a hard-bake step when etching Parylene through photoresist masks. If a hard-bake is required, or if resist is intended for use as a sacrificial structure, we recommend a lengthy hard-bake at low (<100 °C) temperature under an inert atmosphere to minimize thermal stress and oxidation.

### 3.5. Photoresist Stripping

**Figure 18:**
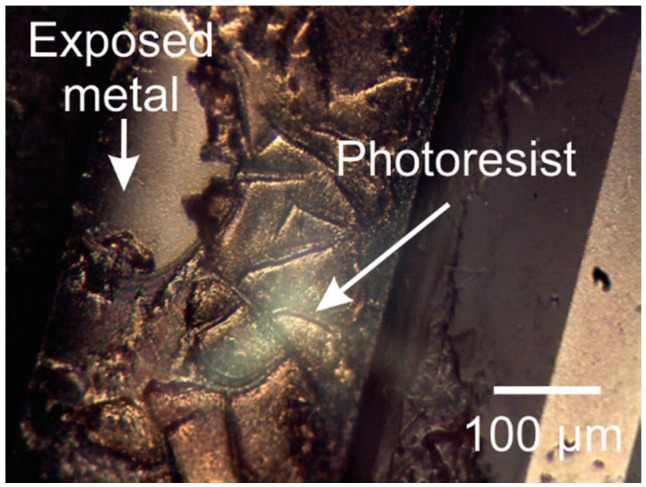
Oxygen plasma treated AZ P4620 photoresist residue on metal feature.

Generally, photoresists (whether a mask or sacrificial layer) are removed with acetone or other organic solvents. Inorganic solvents, such as sulfuric acid, are used when photoresist cannot be easily removed with organic solvents alone. Oxygen plasma, a dry method, is also used to remove photoresist masks but is often impractical as it removes Parylene at the same rate. Figure 18 shows residual AZ P4620 photoresist from the etch mask on a platinum electrode after acetone soaking.

#### 3.5.1. Sacrificial Layer

Stripping of sacrificial photoresist used to define features such as channels in Parylene devices can be particularly challenging after O_2_ DRIE process. For example, in our experience, long soak duration in acetone (30 h at room temperature) was required to remove sacrificial photoresist used to define a microfluidic channel. Such lengthy soaks may lead to delamination of weakly adhered Parylene layers. To address both the length soak time and preservation of device integrity, several parameters were evaluated to accelerate dissolution of AZ P4620 used as a sacrificial layer for microfluidic channels (26.3 mm in length [48]). The combination of increased acetone volume, heating (40 °C) and agitation (magnetic stir-bar) significantly reduced soak time, mitigating risk of Parylene delamination and reducing clearance time.

#### 3.5.2. Oxygen Plasma Exposed Photoresist

**Figure 19:**
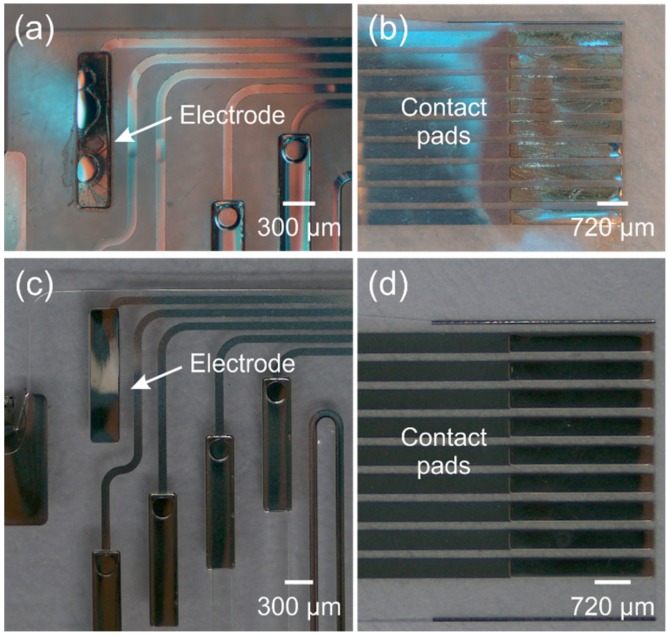
DRIE oxygen plasma exposed AZ P4620 photoresist residue on (**a**) a platinum electrode; and (**b**) contact pads. Cleaned (**c**) platinum electrode; and (**d**) contact pads with homemade stripper.

Photoresist subjected to prolonged oxygen plasma can become crosslinked and difficult to strip using acetone alone (Figure 19a,b). This necessitates the use of more aggressive strippers. Several solutions were evaluated to remove this photoresist from platinum electrodes and contact pads but only piranha solution and homemade stripper successfully removed any residual photoresist.

Upon mixing the Piranha solution (4 parts sulfuric acid (H_2_SO_4_) and 1 part 30% hydrogen peroxide (H_2_O_2_)), an exothermic reaction occurs that raises the solution temperature above 100 °C. Soaking the Parylene device in a piranha solution at temperatures above 60 °C resulted in metal delamination. Consequently, devices were soaked only after the solution cooled down to 40 °C for 3 min. Although piranha was successful, evidence that Parylene was partially oxidized after thermal treatment suggests that this method is too aggressive for Parylene-based devices. On the other hand, the homemade stripper, consisting of 1 part Remover PG (*N*-methyl-2-pyrrolidone based) and 1 part of AZ 726 (<3% tetramethylammonium hydroxide based developer), successfully removed photoresist residue after a 15-min soak at 50 °C as shown in Figure 19c,d.

### 3.6. Release

Releasing the device from the support or carrier wafer is the final step in Parylene MEMS processing. Most commonly, the support is a silicon wafer with a thin native oxide layer and the Parylene adhesion to this material is quite weak. In such cases, Parylene devices can be simply peeled off using tweezers, or the wafer can be submerged in water and the devices allowed to float to the surface. In some cases, however, the adhesion between Parylene and the support can be very strong and releasing Parylene without destroying the thin-film devices can be nearly impossible. In many cases this adhesion is deliberate; sacrificial adhesion layers such as aluminum [45,118] or chrome [85] have been used for complex Parylene processing and devices are then released using KOH or anodic NaCl [45,118] etching for Al, or chrome etchant for Cr [85]. We also demonstrated the release of Parylene devices from a support substrate of polyethylene terephthalate (PET) by boiling the ensemble in water for several minutes. In other scenarios, the adhesion may be unintentional or undesirable. We observed that Parylene devices annealed *on wafer* at 200 °C for 48 h under vacuum and became virtually impossible to remove. In such situations, the only remaining option may be to etch away the underlying silicon or other carrier substrate.

## 4. Discussion

Applying complex, high-resolution micromachining processes to Parylene C substrates is possible but will require modification of nearly every protocol developed for silicon or other rigid substrates. No single definitive set of guidelines exists but we can describe a useful compilation of best practices.

Efforts should be taken to minimize exposure to heat during every step of the fabrication process. Avoiding the glass transition temperature (90 °C) of Parylene is typically unrealistic but avoiding or minimizing the use of high temperature (>100 °C) processes can prevent oxidization, thermal stress, the formation of gas bubbles, or irreversible changes to Parylene morphology. High temperature anneals (200 °C for 48 h in vacuum) are useful to improve moisture barrier properties or to shape Parylene by exploiting thermoplasticity, but must be done under vacuum or inert atmosphere and Parylene must be allowed to cool slowly to room temperatures over several hours. Rapid heating above 120 °C on a hotplate or during PVD can introduce destructive stress and must be avoided.

Parylene C is broadly amenable to most photolithography techniques, including electron-beam lithography but users should be aware of the risk of oxidation due to radiation exposure and complications from gas transport in Parylene films arising from off-gassing of thick photoresist films. Avoiding the use of a hard-bake following development, removing thick edge beads and avoiding high intensity or long duration UV exposure, are all recommended practices. Patterned photoresist masks have been used with success to transfer high-aspect ratio structures to Parylene substrates using O_2_ RIE and DRIE but this method requires very thick photoresist films due to low selectivity during O_2_ etching.

Metal deposition onto Parylene films can prove incredibly challenging. Evaporative PVD can introduce thermal stress and cracking of either the Parylene or metal structures, while sputtering can introduce film-stress which can warp or wrinkle the Parylene film. These challenges are exacerbated by high-melting point metals and thicker metal films. The use of cooled stages or heat-sinking may help during evaporative PVD but the insulative nature of Parylene coatings makes such thermal control difficult. We have observed the best results by using an e-beam deposition system with a throw distance (distance between target and metal crucible) greater than 20″ and splitting depositions of thick films into multiple steps punctuated with 15 min pauses.

Finally, for those Parylene-based bioMEMS intended for chronic use under wet-saline environment, we note that the risk of water permeation or delamination remains one of the largest obstacles. We observed WVTR decreasing significantly, for films thicker than 5 µm, following a 48 h thermal anneal and this is consistent with various literature and anecdotal reports about the advantages of annealing Parylene devices and the difficulties using very thin Parylene as a moisture barrier. The large variation in WVTR measurements between different sources prompts a call for more research into how deposition parameters change barrier properties. New methods to improve Parylene adhesion to thin-film metal would similarly help increase Parylene-device lifetime in vivo.

## 5. Conclusions

In conclusion, while applying micromachining to Parylene substrates can be challenging, with attention to the limited thermal budget and polymeric properties of Parylene, most processes can be successfully transferred to this thin, flexible material. We believe that the observations and recommendations we list here can serve as the basis for a series of best-practices regarding Parylene microfabrication. Continuing research is needed, particularly on the adhesion and barrier properties in the construction of multi-layer devices. By solving these and other challenges Parylene stands to become a key material in a new generation of flexible electronic microdevices and polymer MEMS.

## Figures and Tables

**Table 1:** Water vapor transmission rate (WVTR) measurements for different un-annealed and annealed Parylene film thickness.

Reference		Thickness (µm)	Mean WVTR (g·mm/m^2^·day)	Standard Error	Temperature and Relative Humidity (Mean ± SE)
This work	Un-annealed	5	0.0194	0.0012	20.6 ± 0.14 °C, 34 ± 3%
		10	0.0176	0.0010	20.7 ± 0.13 °C, 32 ± 3%
		15	0.0185	0.0025	20.8 ± 0.12 °C, 33 ± 3%
	Annealed	5	0.0185	0.0014	20.6 ± 0.14 °C, 33 ± 3%
		10	0.0128	0.0010	20.7 ± 0.14 °C, 34 ± 3%
		15	0.0139	0.0024	20.7 ± 0.13 °C, 35 ± 3%
Specialty Coating Systems		-	0.0830	-	37 °C, 90% ASTM F1249
Para Tech		-	0.0550	-	37 °C, 90% ASTM F1249
Menon et al., 2009	Un-annealed	9	0.0547	-	20 °C, 30% ASTM D1653
	Annealed	9	0.0276	-	

**Table 2:** Sterilization methods used and their effect on Parylene. “No adverse effects recorded” indicates the sterilization method was used in literature but no adverse effects on adhesion or bulk properties was recorded, while “n/a” indicates no use of the sterilization method was found in literature.

Sterilization Method	Effect on Bulk Parylene	Effect on Parylene-Parylene Adhesion	Effect on Parylene to Metal Adhesion	Effect on Parylene Adhesion to Other Materials	Reference
Electron beam ISO 11137	Chemical structure changed: partial breakage of C-Cl bonds, ionization of polymer, crystallinity decrease	No adverse effects recorded	No adverse effects recorded	n/a	[92,93]
Gamma radiation ISO 11137	Recombination, cross-linking (increases strength and decreases elongation), loss in bond strength	n/a	Decreased adhesion, causing loss of electrical insulation capabilities	To silicon wafer: Crystallinity increased, no change in Young’s modulus	[91,94]
Ethylene oxide ISO 11135	Formation of inorganic chlorides, reduction of chlorine amount. EtO is toxic, carcinogenic, flammable, explosive	For sterilization after thermal annealing, no adverse effects recorded	Decreased electrical insulation capabilities (but not as damaging as gamma sterilization	To glass: No adverse effects recorded	[93,95,96]
Autoclave (steam) ISO 17665	Parylene became brittle and hard, decreased adhesion, changed chemical stability, did not contaminate	Decreased adhesion	Decreased adhesion	To silicon: Significant decrease in adhesion, Parylene crystallinity increased, electrical stability decreased	[57,91,97,98,99]
H_2_O_2_ Plasma ISO/NP 22441 *	No change in adhesion. Recommended “best suitability for Parylene” (based on XRD testing), successfully killed bacteria without degradation of Parylene coating	n/a	n/a	To Silastic: Parylene withstood implantation and was unaffected. However, coating was easily peeled off after implantation, suggesting poor Parylene-Silastic adhesion	[93,100,101]
Antibiotic coating No standard	0.5–0.75 mg/mL concentration of tetracycline nanoparticles completely eradicated *E. coli* but not aureus bacteria	n/a	n/a	n/a	[102]

* NP: the standard is a new proposal and is still under consideration by the ISO.

**Table 3:** Parameters for photoresist spin curves on different surfaces.

	AZ P4620	AZ 5214E-IR
Surfaces	Silicon, Parylene-coated silicon, glass (100 mm wafers)	
Pre-spin	500 rpm for 5 s	500 rpm for 8 s
Spin acceleration	~1000 rpm/sec	
Main spin	1000, 2000, 3000, 4000, 5000 rpm for 45 s	
Bake	5 min at 90 °C	70 s at 90 °C
